# Targeted Lipidomics Reveal the Effect of Perchlorate on Lipid Profiles in Liver of High-Fat Diet Mice

**DOI:** 10.3389/fnut.2022.837601

**Published:** 2022-03-14

**Authors:** Qiao Wang, Wanying Song, Yimei Tian, Peihao Hu, Xin Liu, Lin Xu, Zhiyong Gong

**Affiliations:** Key Laboratory for Deep Processing of Major Grain and Oil (The Chinese Ministry of Education), College of Food Science and Engineering, Wuhan Polytechnic University, Wuhan, China

**Keywords:** perchlorate, lipid metabolism, high-fat diet, targeted lipidomics, C57BL/6J mice

## Abstract

Perchlorate, commonly available in drinking water and food, acts on the iodine uptake by the thyroid affecting lipid metabolism. High-fat diets leading to various health problems continually raise public concern. In the present study, liver lipid metabolism profiles and metabolic pathways were investigated in C57BL/6J mice chronically exposed to perchlorate using targeted metabolomics. Mice were fed a high-fat diet and treated orally with perchlorate at 0.1 mg/kg bw (body weight), 1 mg/kg bw and 10 mg/kg bw daily for 12 weeks. Perchlorate induced disorders of lipid metabolism *in vivo* and hepatic lipid accumulation confirmed by serum biochemical parameters and histopathological examination. There were 34 kinds of lipid in liver detected by UHPLC-MS/MS and key metabolites were identified by multivariate statistical analysis evaluated with VIP > 1, *p*-value < 0.05, fold change > 1.2 or < 0.8. Perchlorate low, medium and high dose groups were identified with 11, 7 and 8 significantly altered lipid metabolites compared to the control group, respectively. The results of the metabolic pathway analysis revealed that the differential metabolites classified into different experimental groups contribute to the glycerophospholipid metabolic pathway. These findings provide insights into the effects of perchlorate on lipid metabolism during long-term exposure to high-fat diets and contribute to the evaluation of perchlorate liver toxic mechanisms and health effects.

## Introduction

Perchlorate, considered a persistent inorganic pollutant, is an anionic compound usually combined with potassium, sodium or ammonium (1). The presence of perchlorate in the environment is mainly through natural formation and artificial chemical synthesis, and this perchlorate contaminates the water and enters the food chain through plant absorption and enrichment (2). Since the 1990s, perchlorate has been found to be widely present in drinking water and foods which has been raised as a public health concern (3). Numerous studies have reported high detection frequencies of perchlorate in foods such as vegetables, fruits, milk and other foods in amounts deserving of attention (4–6). Approximately 83% of perchlorate is exposed to humans through foods (7). Upon entry into the body, perchlorate competitively inhibits iodine uptake by the thyroid and disrupts the synthesis of thyroid hormones (8), which play a pivotal role in regulating of lipid metabolism (9).

With the improvement of human nutrition, excessive fat intake is considered as a persistent public health problem. Long-term high-fat diet (HFD) can lead to weight gain and a series of adverse reaction, including obesity, hepatic steatosis, hyperlipidemia and hyperglycemia (10, 11). In this case, toxicants contribute to oxidative stress *in vivo*, leading to further abnormalities in hepatic lipid metabolism. Experimentally, it has been shown that the administration of harmful substances (endotoxins or pro-oxidants) to obese mice can lead to severe liver damage and even death (12, 13).

In rodent studies, perchlorate interferes with endocrine function and causes disruptions in lipid metabolism, which in turn causes fat accumulation, weight gain, insulin resistance and abnormal glucose tolerance (14). Lipids have great biological importance in physiological activities such as forming cell membranes, storing energy and transmitting cellular signals, thus lipids largely reflect the metabolic status between health and disease (15). Disorders of lipid metabolism may lead to a range of diseases such as diabetes, obesity, atherosclerosis, coronary heart disease and brain damage (16). Further the liver is the most activated organ for lipid metabolism, and disorders of lipid metabolism may result in fat enrichment and degeneration within hepatocytes. Lipids of interest include phospholipids, cholesterol, sphingolipid and triglycerides.

To our knowledge, there is little published information on the chronic effects of perchlorate on hepatic lipid profiles following a high-fat diet. Studies using lipidomics to explore endocrine disruption by perchlorate have not been reported, but some studies suggest that exposure to exogenous pollutants is an important cause of disruption of lipid metabolism in the body and leads to obesity by affecting metabolic pathways (17). Therefore, the present study applied targeted metabolomics to profile hepatic lipid metabolism in mice with long-term perchlorate intervention. The aim of this work was to investigate the mechanism of hepatotoxicity of perchlorate on lipid metabolism and to contribute to the understanding of the health effects of perchlorate chronically.

## Materials and Methods

### Chemicals and Reagents

Sodium perchlorate (NaClO_4_) standard aqueous solution (10 mg/L) was purchased from O2SI Smart Solution (Charleston, SC, USA). All lipid standards and internal standards were purchased from Avanti Polar Lipids Inc. (Alabaster, AL, USA). Formic acid (≥95%) and chloroform were obtained from Sigma-Aldrich (St. Louis, MO, USA). Acetonitrile, methanol, isopropanol, *n*-Hexane were all HPLC grade, obtained from Thermo Fisher Scientific (Waltham, MA, USA). Sodium chloride, sodium sulfate and sodium hydroxide were supplied by Sinopharm Chemical Reagents Co., Ltd.

### Animal Experiment

A total of 24 6-week-old male C57BL/6J mice (bw: 27–29 g) were purchased from Hubei Province Animal Research Center, Hubei, China. The study was approved by the Institutional Animal Care and Use Committee Tongji Medical College, Huazhong University of Science and Technology (IACUC Number: 2395).

All mice were housed in a specific-pathogen-free animal laboratory with a 12/12 h light-dark cycle (temperature 24 ± 1°C; relative humidity 55–65%). After 3 days of acclimatization, animals were randomly divided into one control group and three experimental groups of six mice each: low-dose group (LG, 0.1 mg/kg bw daily), middle-dose group (MG, 1 mg/kg bw daily), high-dose group (HG, 10 mg/kg bw daily) and control group (CG, received an equivalent volume of saline). Sodium perchlorate was dissolved in physiological saline and administered orally to the mice. High-fat feed and drinking water were supplied *ad libitum*, the high-fat feed consisted of 10.0% fat, 15.0% protein, 30.6% starch, 4.6% ash and 6.6% moisture. Mice were routinely fed for 12 consecutive weeks and weighed every 6 days. Blood samples and liver tissues were harvest after all mice sacrifice via cervical dislocation, liver weight and body weight were recorded. Liver coefficients were calculated according to the formula: Liver coefficient (%) = liver weight (g) / body weight (g) × 100%. All collected samples were stored at −80°C until assayed.

### Determination of Biochemical Parameters

The serum of mice was separated from whole blood by centrifugation and measured using commercial ELISA kits (Nanjing Jiancheng Bioengineering Institute, China), to assess the serum biochemistry. Parameters included the liver function markers aspartate aminotransferase (AST), alanine aminotransferase (ALT), malondialdehyde (MDA); and indicators of lipid metabolism, total cholesterol (TC), total triglyceride (TG), high-density lipoprotein cholesterol (HDL-c), and low-density lipoprotein cholesterol (LDL-c).

### Histological Analysis of Liver Section

Liver tissues were removed after the mice were sacrificed and fixed with 4% paraformaldehyde, embedded in paraffin, and processed for hematoxylin and eosin (H&E) staining using a standard protocol (18). The sections were observed and photographed with an inverted microscope at 400 × magnification.

### Pretreatment of Lipid Analysis

The pretreatment of liver was performed as previously described (19), with some modifications. 100 mg liver tissue sample was placed in a homogenate tube thawed on ice, 1 mL ice water was added and homogenized for 8 min. Then 0.1 mL homogenate was mixed with 1.2 mL methanol/chloroform (2:1, v/v, 0.1% MTBE) and 0.6 mL water vortexed for 1 min in 2 mL EP tube. The mixture was sonicated for 5 min and centrifuged at 10,000 g for 10 min at 4°C. The supernatant was collected and dried with nitrogen gas. Finally, the residue was reconstituted in 1 mL initial mobile phase and filtered through a 0.22 μm membrane for UHPLC-MS/MS analysis.

Quality control (QC) sample was prepared by mixing equal amounts (50 μL) of each extracted sample (24 in total). Moreover, the QC specimen was analyzed every six samples throughout the whole analysis procedure.

### UPLC-MS/MS Analysis

Ultra-high performance liquid chromatography-tandem mass spectrometry analysis was performed as previously described (20). Briefly, UHPLC-MS/MS analysis was undertaken on QTRAP^®^ 6,500+ LC-MS/MS system (AB Sciex, USA) equipped with Acquity HSS T3 column (2.1 × 100 mm i.d., 1.8 μm, Waters, USA). The mobile phase was composed of water/methanol/acetonitrile (1/1/1, v/v/v) containing 10 mM ammonium formate and 0.1% formic acid (solvent A) and isopropanol (solvent B) at a flow speed of 0.4 mL/min. The gradient elution program was set as follows: solvent A initially at 80% declined to 10% within 12 min and held for 1 min, then decreased to 80% within 2 min and held for 1 min. The column temperature maintained at 45 °C and the injection volume was 10 μL.

Initially separated liver samples were scanned in a triple quadrupole containing ion trap. The system is equipped with ESI Turbo ion spray port, which can be operated by Analyst 1.6.3 software. The conditions used for the electrospray source were shown as follows: the ion source temperature, 550 °C; the ion spray voltage, 5,500 V in positive mode (or −4,500 V in negative model); the ion source gas I, 55 psi; the gas II, 55 psi; the curtain gas, 35 psi; the collision gas, medium; quantification of ion pairs corresponding to lipids by multiple reaction monitoring mode (MRM). Mass spectrometry information of the target lipid metabolites, including compound name, MRM transition, optimized dephasing potential (DP) and collision energy (CE) is shown in Supplementary Table 1. All targets were quantified separately using D31-PG(16:0/18:1), D31-PE(16:0/18:1), D31-PC(16:0/18:1), C17-Sphinganine and D5-TAG(17:0/17:1/17:0) as internal standards to quantify phosphatidylglycerol (PG), Lyso-phosphatidylcholine (LPC), phosphatidylcholine (PC), phosphatidylethanolamine (PE), sphingolipids (SM) and triglyceride (TAG), respectively.

### Raw Data Processing and Metabolic Pathway Analysis

Raw mass-spectrometry data were processed with Software Analyst 1.6.3. The pooled sample was used as quality control (QC) to validate the reproducibility and stability of lipid metabolites in the positive and negative ion mode of the method and equipment. The quantification of target lipid metabolites was based on signal intensity relative to the corresponding internal standard by the multiple reaction monitoring mode (MRM) of triple quadrupole mass spectrometry. The integration and calibration of chromatographic peaks was performed with Multiquant Software version 3.0.2 (AB Sciex Inc.).

Quantitative data of lipid metabolites were analyzed by SIMCA-P software version 14.1 (Umetrics, Sweden) using an orthogonal partial least squares discriminant analysis (OPLS-DA) model for differential analysis to screen for potentially differential lipids. Lipid metabolites with VIP value > 1, *p*-value < 0.05 and FC > 1.2 or FC < 0.8 were defined as significantly different. Metabolic pathway analysis was performed with Metaboanalyst 5.0 (www.metaboanalyst.ca).

### Statistical Analysis

All data were collected and computed into IBM SPSS Statistics 19 and expressed as mean ± standard deviation (SD). The difference between the experimental groups and the control group was highly significant by one-way ANOVA. *P*-values < 0.05 were considered statistically significant.

## Results and Discussion

### Body Weight and Liver Coefficient

**Figure 1:**
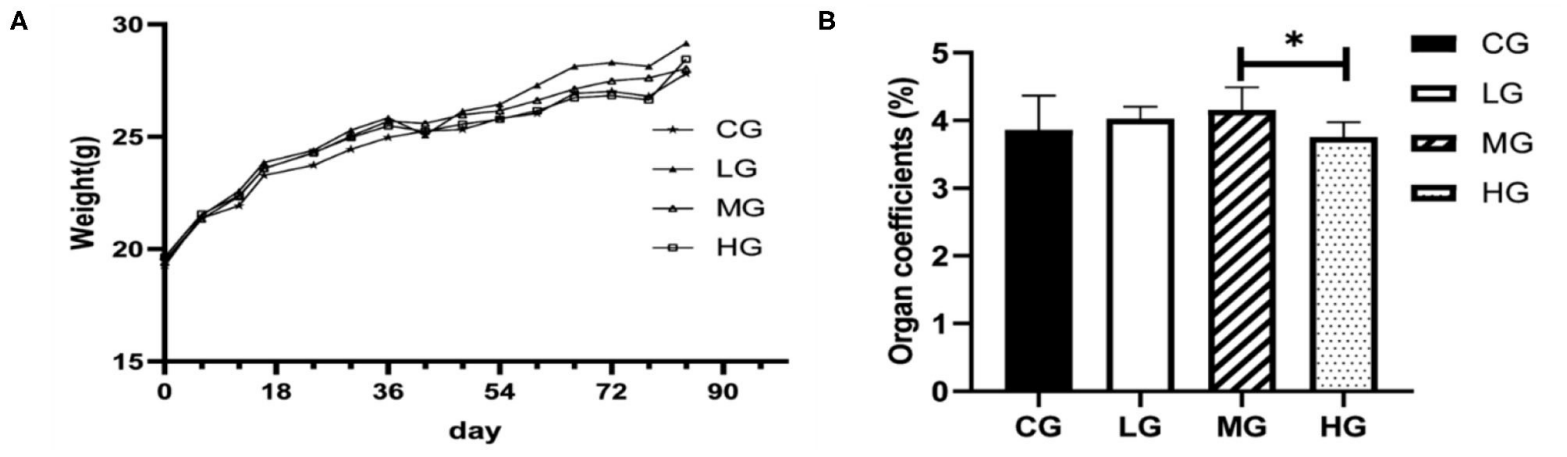
Changes of body weights of the mice during the experiment **(A)**, liver coefficient **(B)** (*P < 0.05, *n* = 6).

The weights of the mice were recorded every 6 days for total of 12 weeks, and fresh livers were weighed immediately upon harvest, individual ratio of liver to body weights were calculated. Figure 1 shows changes in body weight and liver coefficient. Body weight trended to increase gradually over perchlorate exposure time. Within 1 week after the start of the experiment, there was almost no change in body weight between the groups, and the difference gradually appeared after 1 week, indicating that short-term exposure to perchlorate had less effect on the growth of mice, and this result was consistent with the previously reported (21). Compared with the CG group, the body weight of the LG group was significantly increased (*p* < 0.05) at the end of the experiment, but there was no significant difference in MG and HG groups (Figure 1A). No significant differences were observed in liver coefficient between treatment groups and control group, whereas HG group showed a significantly decreased (*p* < 0.05), after mice were exposed to different doses of perchlorate (Figure 1B). These results suggest that chronic high dose intake of perchlorate might have affected the liver coefficients.

### Biochemical Parameters

The seven biochemical indicators in serum were divided into two subgroups according to their physiological meaning (Table 1). AST, ALT and MDA as indicators of liver function, there was no significant difference between the perchlorate exposed groups and the control group. With the increase of perchlorate dose, the content of MDA increases correspondingly. MDA is generated by lipid peroxidation, so the change in MDA content reflects the extent of membrane damage (22). The lipid metabolic markers include serum TC, TG, HDL-c, LDL-c. Compared with those of the CG group, TC in MG and HG groups showed markedly increased, TG in HG group was increased and the difference was statistically. The high-fat diet resulted in elevated serum levels of TC, TG and LDL cholesterol and reduced levels of HDL cholesterol in mice (23). However, under the influence of perchlorate, LDL cholesterol levels did not change significantly, and HDL levels increased significantly in the experimental groups, which indicated that perchlorate caused lipid metabolism disturbance.

**Table 1:** Levels of serum biochemistry parameters in mice from experiment and control groups.

**Parameter**	**CG**	**LG**	**MG**	**HG**
AST (U/L)	26.75 ± 4.50	26.00 ± 1.63	25.75 ± 1.89	28.75 ± 2.06
ALT (U/L)	11.00 ± 0.82	10.50 ± 2.08	11.00 ± 3.16	11.50 ± 1.73
MDA (nmol/mL)	4.94 ± 1.77	5.19 ± 0.98	5.96 ± 2.32	6.47 ± 1.10
TC (mmol/L)	1.19 ± 0.11^**a**^	1.284 ± 0.04^**a, b**^	1.42 ± 0.13^**b**^	1.464 ± 0.23^**b**^
TG (mmol/L)	0.12 ± 0.03^**a**^	0.12 ± 0.01^**a**^	0.20 ± 0.05^**a, b**^	0.17 ± 0.23^**b**^
HDL-c (mmol/L)	1.21 ± 0.10^**a**^	1.27 ± 0.04^**a, b**^	1.35 ± 0.12^**a, b**^	1.49 ± 0.25^**b**^
LDL-c (mmol/L)	0.17 ± 0.04	0.20 ± 0.04	0.25 ± 0.07	0.18 ± 0.03

*Mean ± standard deviation values in the same row for each parameters followed by different letters are significantly different (p < 0.05)*.

### Histopathology Examination

**Figure 2:**
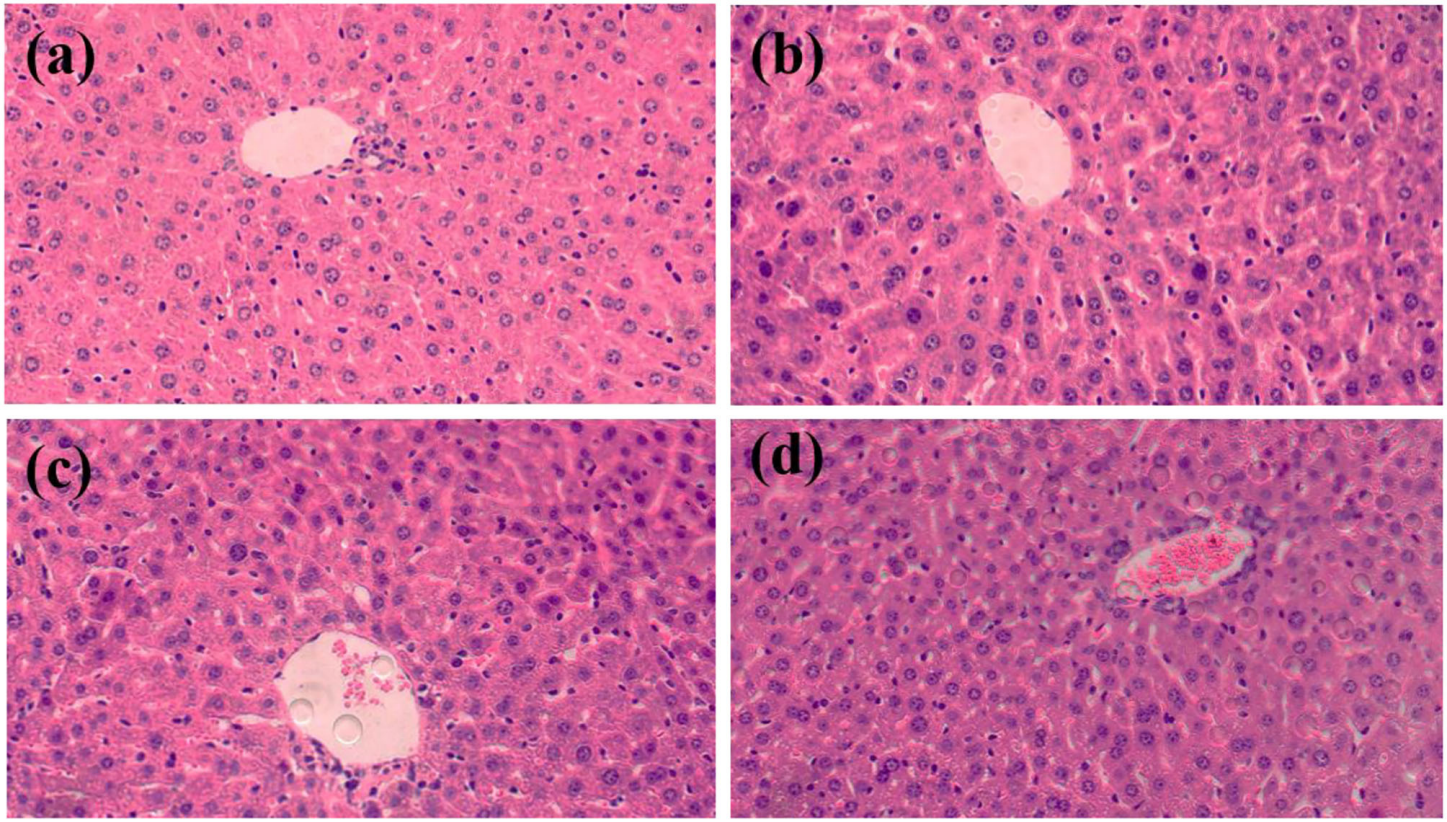
Histopathological analysis of liver (HE, 400×). **(A)** CG, **(B)** LG, **(C)** MG, and **(D)** HG.

Histopathological analysis was performed after oral administration of treatments in C57BL/6J mice for 12 weeks. Liver sections with H&E staining were imaged using an inverted fluorescence microscope (Figure 2). In the 400X magnification images, vacuolization was observed in the liver sections of all groups. Small amounts of inflammatory cells were present in the CG and LG groups. In the MG group, inflammatory cells infiltrate of liver lobules and dilated congested central vein were observed. The same was more evident in the HG group. Combined with the results of serum biochemical parameters, it further demonstrates that perchlorate affects lipid accumulation and lipid metabolism disorders in the liver *in vivo*, which may be associated with inflammation, obesity and other chronic diseases (24–26).

### Quantification of Liver Lipid Metabolites

**Figure 3:**
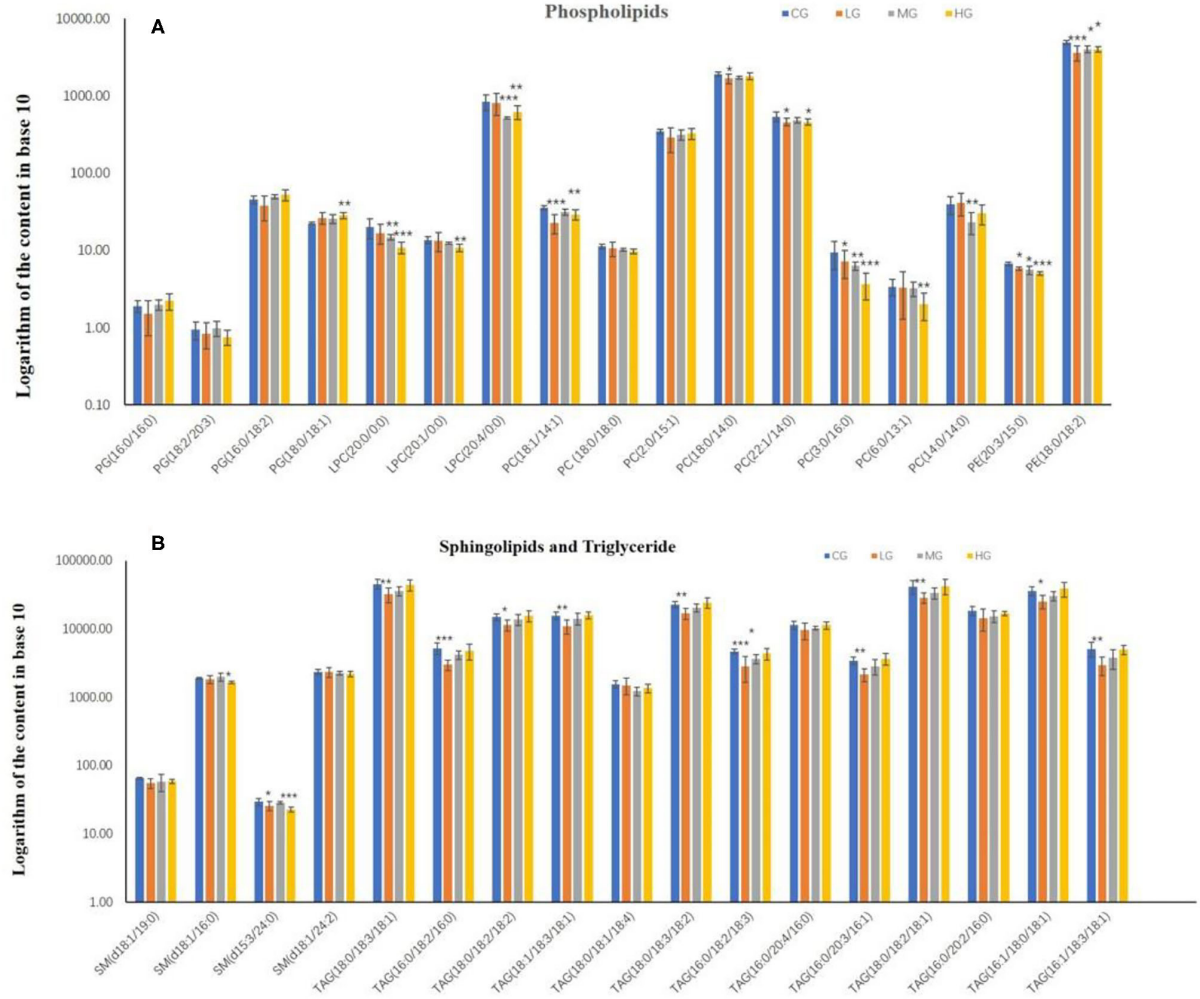
The content of phospholipids **(A)** and sphingolipids and triglyceride **(B)** in the liver of mice in high-dose group (HG), middle-dose group (MG), low-dose group (LG) and control group (CG) (^*^P < 0.05, ^**^P < 0.01, ^***^P < 0.001, compared with the control group, *n* = 6).

To investigate the effect of perchlorate on hepatic lipid metabolism in mice fed a high-fat diet, 34 common lipids were selected as targets for quantitative. These included 4 phosphatidylglycerols (PG), 8 phosphatidylcholines (PC), 3 lyso-phosphatidylcholines (LPC), 2 phosphatidylethanolamines (PE), 4 sphingolipids (SM) and 13 triglycerides (TAG). All mice liver samples were subjected to UHPLC-MS/MS analysis. Quantification of PGs, PCs, LPCs, PEs, SMs and TAGs by D31-PG (16:0/18:1), D31-PC (16:0/18:1), D31-PE (16:0/18:1), C17-sphinganine and D5-TAG (17:0/17:0/17:0), respectively. The relative standards deviation values of peak areas of lipids in all QC samples are required to be <10%. The content of all lipids in the liver of C57BL/6J mice is taken as a logarithm with a base of 10, as shown in the Figure 3.

In phospholipids metabolism (Figure 3A), PG (16:0/16:0), PG (16:0/18:2), PG (18:0/18:1), PC (2:0/15:1) and PC (18:0/14:0) shown an increasing trend in the experimental groups with dose-effect relationship. PC (18:0/14:0) in the LG group was significantly decreased compared to the CG group (*p* < 0.05), and PG (18:0/18:1) in the HG group was very significantly elevated compared to the CG group (*p* < 0.01). However, LPC (20:0/0:0), LPC (20:1/0:0), PC (3:0/16:0), PC (6:0/13:1) and PE (20:3/15:0) formed a decreasing trend among the experimental groups, and all of them were significantly reduced in the HG groups compared to the CG group (*p* < 0.01), with PC (3:0/16:0) and PE (20:3/15:0) showing significance in all experimental groups and LPC (20:0/0:0) showing significance in the MG group (*p* < 0.01). Moreover, PC (18:1/14:1), PC (22:1/14:1) and PE (18:0/18:2) were decreased in experimental groups compared to the CG group, with the exception of PC (18:1/14:1) and PC (22:1/14:0) in the MG group, which showed significant differences in all groups (*p* < 0.05).

In sphingolipids and triglyceride metabolism (Figure 3B), 4 types of sphingolipids were lowered in the experimental groups in comparison with the CG group, with significant differences both in the HG group for SM (d18:1/16:0) and in the LG and HG groups for SM (d15:3/24:0). This result could indicate that the intake of perchlorate in high-fat dietary mice may reduce sphingolipids. Another aspect is that all triglyceride lipids showed an increasing trend in the experimental groups except for TAG (18:0/18:1/18:4). Furthermore, in contrast to the CG group, TAG (18:0/18:3/18:1), TAG (16:0/18:2/16:0), TAG (18:0/18:2/18:2), TAG (18:1/18:3/18:1), TAG (18:0/18:3/18:2), TAG (16:0/20:3/16:1), TAG (18:0/18:2/18:1), TAG (16:1/18:0/18:1) and TAG (16:1/18:3/18:1) significantly decreased in LG group, as well as TAG (16:0/18:2/18:3) in the LG and MG groups.

PC and PE account for more than 50% of the total phospholipid species in eukaryotic membranes and play a major role in the structure and function of biological membranes (27). PE is synthesized *in vivo* by methylation to 1-methylphosphatidylethanolamine (PMME), which is then methylated to produce dimethyl phosphatidylethanolamine (PDME), and PDME is synthesized under the action of enzymes to PC (28). The phospholipase PLA2 hydrolyzes PC to LPC, and LPC and PC can be interconverted (29). It has been demonstrated that PC mediates the promotion of cell proliferation, growth and programmed cell death (30). LPC is a pro-inflammatory mediator that promotes inflammation in acute injuries or chronic diseases (31). One study found that high-fat diet-induced obesity decreased LPC levels in the body (32), which is consistent with the reduction of LPC levels with increasing perchlorate levels in the present study, showing that perchlorate exacerbates the symptoms of high-fat diet-induced obesity. PGs, a surface-active lipid *in vivo*, showed an increasing trend in our study, implying that the increased dose of perchlorate exposure may promote the formation of phosphatidylglycerols. Triglycerides are closely related to metabolic diseases such as atherosclerosis, hyperlipidemia and obesity (33, 34). Most of the triglycerides in the experiment showed significant changes reflecting that the metabolic homeostasis of high-fat mice was disturbed by perchlorate intake, with a dose-effect relationship.

### Metabolite Screening

Hepatic lipid metabolism is influenced by multiple factors, analysis of variable importance is carried out as a key step in the profiling analysis of the effects of perchlorate on lipid metabolism in mice fed a high-fat diet. To this end, the study analyzed the differential metabolites by establishing the OPLS-DA model. Different experimental groups were examined in comparison with the control group, and differential lipid metabolites were identified for VIP values > 1, statistically significant *P* values < 0.05 and fold change > 1.2 or <0.8 (35). A total of 21 lipid metabolites were identified among the experimental groups, including 10 types of phospholipids, 1 type of sphingolipid and 10 types of triglycerides. In addition, 11, 7 and 8 differential lipid metabolites were identified in the LG, MG and HG groups, respectively (Table 2). Also, TAG(16:0/18:2/18:3) and TAG(18:0/18:3/18:1) were selected in both the LG and MG groups, and LPC(20:4/0:0), LPC(20:0/0:0) and PC(3:0/16:0) were selected in both the MG and HG groups. By the results, the differential lipid metabolites were primarily phospholipids and triglycerides, as well as the lipids identified were mostly different in the three experimental groups. It can be inferred that perchlorate mainly affected phospholipid metabolic pathway and triglyceride metabolic pathway, and that mice may be more sensitive to perchlorate dose in long-term lipid metabolism. It is noteworthy that in the above results total serum triglycerides were significantly increased in the experimental groups, which is consistent with other environmental pollutants (e.g., perfluorinated compounds, BPA, PCBs, etc.) that affect the lipid metabolism leading to fat accumulation (14), while the identified triglycerides in the liver were down-regulated. This may be due to the effect of high-fat diet on the liver by perchlorate, which in turn reduces total lipid accumulation in the liver by accordingly diminishing the hepatic triglyceride concentration as a protection action (36). The mechanism of this result deserve more work for further study.

**Table 2:** Potential differential metabolites identified significantly different in liver in each group.

**Group**	**Compound name**	**VIP**	***P*-value**	**FC**	**Regulation**
LG	PC (18:1/14:1)	1.35	0.000	0.63	Down
	PE (18:0/18:2)	1.31	0.000	0.74	Down
	TAG (16:0/18:2/18:3)	1.30	0.000	0.60	Down
	TAG (16:1/18:3/18:1)	1.27	0.000	0.58	Down
	TAG (16:0/20:3/16:1)	1.24	0.002	0.63	Down
	TAG (18:0/18:3/18:2)	1.20	0.004	0.74	Down
	TAG (18:0/18:2/18:2)	1.17	0.014	0.76	Down
	TAG (18:1/18:3/18:1)	1.16	0.002	0.70	Down
	TAG (16:0/18:2/16:0)	1.16	0.001	0.57	Down
	TAG (16:1/18:0/18:1)	1.09	0.015	0.71	Down
	TAG (18:0/18:3/18:1)	1.07	0.005	0.71	Down
MG	LPC (20:4/0:0)	1.60	0.000	0.62	Down
	LPC (20:0/0:0)	1.58	0.000	0.74	Down
	PC (3:0/16:0)	1.44	0.001	0.67	Down
	TAG (16:0/18:2/18:3)	1.21	0.007	0.77	Down
	PC (14:0/14:0)	1.18	0.005	0.60	Down
	TAG (18:0/18:3/18:1)	1.15	0.035	0.79	Down
	TAG (18:0/18:1/18:4)	1.11	0.006	0.78	Down
HG	LPC (20:0/0:0)	1.60	0.000	0.54	Down
	PE (20:3/15:0)	1.57	0.000	0.75	Down
	PC (3:0/16:0)	1.54	0.000	0.39	Down
	SM (d15:3/24:0)	1.44	0.000	0.77	Down
	PG (18:0/18:1)	1.43	0.001	1.27	Up
	LPC (20:1/0:0)	1.41	0.000	0.79	Down
	PC (6:0/13:1)	1.29	0.001	0.59	Down
	LPC (20:4/0:0)	1.20	0.000	0.74	Down

### Metabolic Pathway Analysis

**Figure 4:**
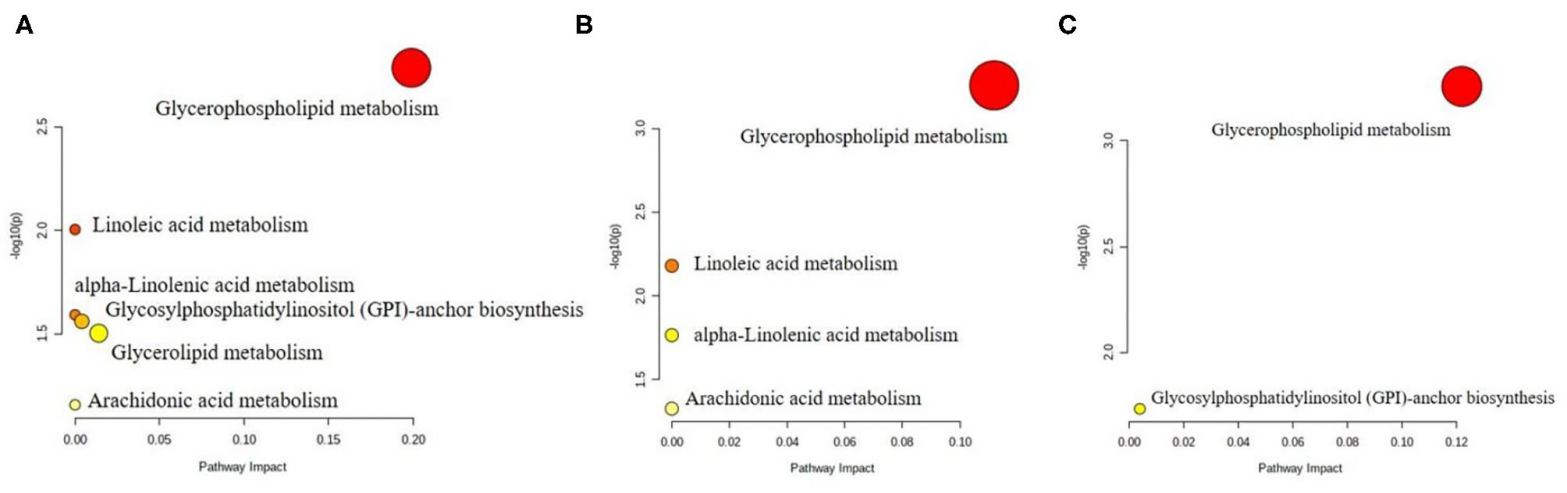
Pathway analysis of differential lipid metabolites in liver from the **(A)** LG group, **(B)** MG group, and **(C)** HG group.

Analysis of the key metabolic pathways included enrichment analysis and topology analysis conducted by Metaboanalyst 5.0 program. Overrepresentation analysis and path topology analysis were tested for hypergeometry and relative intermediacy, respectively. The pathway database was selected from the Rattus norvegicus (rat) in Metaboanalyst 5.0. The path impact on the horizontal axis was the calculated impact value from the path topology analysis, and the –log(p) on the vertical axis was the negative logarithmic shift of the *p*-value calculated in the path enrichment analysis (Figure 4). The pathways analysis revealed a significant enrichment of glycerophospholipid metabolism pathway in all LG, MG and HG groups, suggesting that perchlorate affects the targets of hepatic lipid metabolism in mice fed a high-diet mainly by regulating glycerophospholipid metabolism. In addition, there may be effects on the metabolic pathways such as linoleic acid metabolism, alpha-linolenic acid metabolism, arachidonic acid metabolism and glycosylphosphatidylinositol (GPI)-anchor biosynthesis.

Glycerophospholipids are the most abundant phospholipids *in vivo* and they regulate intracellular molecular signaling pathways via hydrolysis by phospholipases and binding to G protein-coupled receptors on biological membranes, which in turn are involved in various processes such as inflammation, immunity and tumor growth (37). In the above findings, the different glycerophospholipids in the experimental groups contributed higher values in the multivariate analysis, and the changes in lipid metabolic pathways in high-fat mice chronically under the influence of diverse doses of perchlorate directed to the glycerophospholipid metabolic pathway. The results suggest that the effect of perchlorate on lipid metabolism *in vivo* is mainly in the glycerophospholipid metabolic pathway and that the lipids in the glycerophospholipid pathway regulated by perchlorate at doses of 0.1 mg/kg bw, 1 mg/kg bw and 10 mg/kg bw per day are different.

## Conclusion

In summary, the study investigated lipid metabolism in high-fat C57BL/6J mice chronically exposed to perchlorate. On the basis of growth parameters, serum biochemical parameters, histopathological examination and lipid quantification, long-term perchlorate exposure exhibited effects on disorders of serum and hepatic lipid metabolism and lipid accumulation in the liver of mice. Taking advantage of targeted lipidomics, it was found that perchlorate had a significant effect on glycerophospholipid metabolic pathways in mice on a high-fat diet. Investigators should consider the combined effects of perchlorate on metabolic damage *in vivo* in the future. Other small molecule metabolomics, proteomics, transcriptomics, genomics and molecular biology techniques should be applied to enhance the understanding of the toxicological mechanisms of perchlorate. This study lays the foundation for the identification of the effects of perchlorate on lipid metabolism and metabolic pathways.

## Data Availability Statement

The original contributions presented in the study are included in the article/Supplementary Material, further inquiries can be directed to the corresponding author.

## Ethics Statement

The animal study was reviewed and approved by the Institutional Animal Care and Use Committee Tongji Medical College, Huazhong University of Science and Technology.

## Author Contributions

WS, YT, and PH carried out animal experiment, histopathological examination, and sample processing. XL and LX completed targeted lipidomics analysis. The study was designed by QW and ZG. The manuscript was written by QW and WS. All authors contributed to the article and approved the submitted version.

## Funding

This research was supported by the National Natural Science Foundation of China (No. 32172313), the National Key Research and Development Program of China (No. 2017YFC1600500), and the Open Foundation of Hubei Key Laboratory for Processing and Transformation of Agricultural Products (Wuhan Polytechnic University) (No. 2020HBSQGDKFB04).

## Conflict of Interest

The authors declare that the research was conducted in the absence of any commercial or financial relationships that could be construed as a potential conflict of interest.

## Publisher's Note

All claims expressed in this article are solely those of the authors and do not necessarily represent those of their affiliated organizations, or those of the publisher, the editors and the reviewers. Any product that may be evaluated in this article, or claim that may be made by its manufacturer, is not guaranteed or endorsed by the publisher.

## References

[1] Tian Y Xu H Liu S Fang M Wu Y Gong Z. Study on the bioaccessibility and bioavailability of perchlorate in different food matrices *in vitro*. Food Chem. (2020) 333: 127470. 10.1016/j.foodchem.2020.127470 32653684

[2] Wang Z Forsyth D Lau PY Pelletier L Gaertner D. Estimated dietary exposure of Canadians to perchlorate through the consumption of fruits and vegetables available in Ottawa markets. J Agr Food Chem. (2009) 57: 9250–5. 10.1021/jf901910x 19743859

[3] Urbansky ET. Perchlorate as an environmental contaminant. Environ Sci Pollut Res. (2002) 9: 187–92. 10.1007/BF02987487 12094532

[4] Chang WH Chen HL Lee CC. Dietary exposure assessment to perchlorate in the Taiwanese population: a risk assessment based on the probabilistic approach. Environ Pollut. (2020) 267: 115486. 10.1016/j.envpol.2020.115486 32911335

[5] Lee JW Oh SH Oh JE. Monitoring of perchlorate in diverse foods and its estimated dietary exposure for Korea populations. J Hazard Mater. (2012) 243: 52–8. 10.1016/j.jhazmat.2012.09.037 23116718

[6] Liao Z Cao D Gao Z Zhang S. Occurrence of perchlorate in processed foods manufactured in China. Food Control. (2020) 107:106813. 10.1016/j.foodcont.2019.106813

[7] Oh SH Lee JW Mandy P Oh JE. Analysis and exposure assessment of perchlorate in Korean dairy products with LC-MS/MS. Environ Health Toxicol. (2011) 26:e2011011. 10.5620/eht.2011.26.e2011011 22125772 PMC3214986

[8] Massimo T Aldo P Antonio D Eleonora F Patrizia A Paolo V. Relative potencies and additivity of perchlorate, thiocyanate, nitrate, and iodide on the inhibition of radioactive iodide uptake by the human sodium iodide symporter. Thyroid. (2004) 14: 1012–9. 10.1089/thy.2004.14.1012 15650353

[9] Lee J Ha J Jo K Lim DJ Lee JM Chang SA. High normal range of free thyroxine is associated with decreased triglycerides and with increased high-density lipoprotein cholesterol based on population representative data. J Clin Med. (2019) 8: 758. 10.3390/jcm8060758 31142048 PMC6616420

[10] Liu C Ma J Sun J Cheng C Feng Z Jiang H. Flavonoid-rich extract of paulownia fortunei flowers attenuates diet-induced hyperlipidemia, hepatic steatosis and insulin resistance in obesity mice by AMPK pathway. Nutrients. (2017) 9:959. 10.3390/nu9090959 28867797 PMC5622719

[11] Wang S Moustaid-Moussa N Chen L Mo H Shastri A Su R. Novel insights of dietary polyphenols and obesity. J Nutr Biochem. (2014) 25: 1–18. 10.1016/j.jnutbio.2013.09.001 24314860 PMC3926750

[12] Ito K Kiyosawa N Kumagai K Manabe S Matsunuma N Yamoto T. Molecular mechanism investigation of cycloheximide-induced hepatocyte apoptosis in rat livers by morphological and microarray analysis. Toxicology. (2006) 219: 175–86. 10.1016/j.tox.2005.11.017 16368179

[13] Yang SQ Lin HZ Mandal AK Huang J Diehl AM. Disrupted signaling and inhibited regeneration in obese mice with fatty livers: implications for nonalcoholic fatty liver disease pathophysiology. Hepatology. (2001) 34: 694–706. 10.1053/jhep.2001.28054 11584365

[14] Holtcamp W. Obesogens: an environmental link to obesity. Environ Health Persp. (2014) 120: 62–8. 10.1289/ehp.120-a62 22296745 PMC3279464

[15] Gross RW Han X. Unlocking the complexity of lipids: using lipidomics to identify disease mechanisms, biomarkers and treatment efficacy. Future Lipidol. (2006) 1: 539–47. 10.2217/17460875.1.5.539

[16] Rao MA Hatcher JF Dempsey RJ. Lipids and lipidomics in brain injury and diseases. AAPS PharmSci. (2006) 8:E314. 10.1007/BF02854902 16796382 PMC3231558

[17] Lustig RH. Childhood obesity: behavioral aberration or biochemical drive? Reinterpreting the First Law of Termodynamics. Nat Clin Pract Endocrinol Metab. (2006) 2: 447–58. 10.1038/ncpendmet0220 16932334

[18] Alday-Parejo B Richard F Worthmuller J Rau T Galvan JA Desmedt C. MAGI1, a new potential tumor suppressor gene in estrogen receptor positive breast cancer. Cancers. (2020) 12:223. 10.3390/cancers12010223 31963297 PMC7016640

[19] Liu Y Wang R Zheng K Xin Y Jia S Zhao X. Metabonomics analysis of liver in rats administered with chronic low-dose acrylamide. Xenobiotica. (2020) 50: 894–905. 10.1080/00498254.2020.1714791 31928121

[20] Chen JX Han YS Zhang SQ Li ZB Chen J Yi WJ. Novel therapeutic evaluation biomarkers of lipid metabolism targets in uncomplicated pulmonary tuberculosis patients. Signal Transduct Target Ther. (2021) 6:22. 10.1038/s41392-020-00427-w 33462176 PMC7814055

[21] Yu KO Narayanan L Mattie DR Godfrey RJ Todd PN Sterner TR. The pharmacokinetics of perchlorate and its effect on the hypothalamus-pituitary-thyroid axis in the male rat. Toxicol Appl Pharmacol. (2002) 182:148–59. 10.1006/taap.2002.9432 12140178

[22] Hu Y Liu S Zhu BM. CRISPR/Cas9-induced loss of keap1 enhances anti-oxidation in rat adipose-derived mesenchymal stem cells. Front Neurol. (2019) 10:1311. 10.3389/fneur.2019.01311 32132961 PMC7040357

[23] Yang D Hu C Deng X Bai Y Cao H Guo J. Therapeutic effect of chitooligosaccharide tablets on lipids in high-fat diets induced hyperlipidemic rats. Molecules. (2019) 24:514. 10.3390/molecules24030514 30709014 PMC6385166

[24] Varghese M Griffin C McKernan K Eter L Lanzetta N Agarwal D. Sex differences in inflammatory responses to adipose tissue lipolysis in diet-induced obesity. Endocrinology. (2019) 160:293–312. 10.1210/en.2018-00797 30544158 PMC6330175

[25] Akmatov MK Ermakova T Holstiege J Steffen A Stillfried D Batzing J. Comorbidity profile of patients with concurrent diagnoses of asthma and COPD in Germany. Sci Rep. (2020) 10: 17945. 10.1038/s41598-020-74966-1 33087813 PMC7578650

[26] Ding Y Haks MC Forn-Cuni G He J Nowik N Harms AC. Metabolomic and transcriptomic profiling of adult mice and larval zebrafish leptin mutants reveal a common pattern of changes in metabolites and signaling pathways. Cell Biosci. (2021) 11:126. 10.1186/s13578-021-00642-0 34233759 PMC8265131

[27] Federica G Smith TK. The Kennedy pathway *de novo* synthesis of phosphatidylethanolamine and phosphatidylcholine. IUBMB Life. (2010) 62: 414–28. 10.1002/iub.354 20503434

[28] Haberl EM Pohl R Rein-Fishboeck L Horing M Krautbauer S Liebisch G. Hepatic lipid profile in mice fed a choline-deficient, low-methionine diet resembles human non-alcoholic fatty liver disease. Lipids Health Dis. (2020) 19:250. 10.1186/s12944-020-01425-1 33298075 PMC7727224

[29] Sahu PK Tomar RS. The natural anticancer agent cantharidin alters GPI-anchored protein sorting by targeting Cdc1-mediated remodeling in endoplasmic reticulum. J Biol Chem. (2019) 294:3837–52. 10.1074/jbc.RA118.003890 30659098 PMC6422101

[30] Ridgway ND. The role of phosphatidylcholine and choline metabolites to cell proliferation and survival. Crit Rev Biochem Mol Biol. (2013) 48:20–38. 10.3109/10409238.2012.735643 23350810

[31] Liu-Wu Y Hurt-Camejo E Wiklund O. Lysophosphatidylcholine induces the production of IL-1β by human monocytes. Atherosclerosis. (1998) 137:351–7. 10.1016/S0021-9150(97)00295-5 9622278

[32] Rai S Bhatnagar S. Novel lipidomic biomarkers in hyperlipidemia and cardiovascular diseases: an integrative biology analysis. OMICS. (2017) 21:132–42. 10.1089/omi.2016.0178 28157411

[33] Chaix A Zarrinpar A Miu P Panda S. Time-restricted feeding is a preventative and therapeutic intervention against diverse nutritional challenges. Cell Metab. (2014) 20:991–1005. 10.1016/j.cmet.2014.11.001 25470547 PMC4255155

[34] Tan T Lai CJ Zeng L Liu EH Li P. Enzymatic hydrolysis-based absolute quantification of triacylglycerols in plant oil by use of a single marker. Anal Bioanal Chem. (2014) 406:4921–9. 10.1007/s00216-014-7899-0 24912990

[35] Ou M Li C Tang D Xue W Xu Y Zhu P. Genotyping, generation and proteomic profiling of the first human autosomal dominant osteopetrosis type II-specific induced pluripotent stem cells. Stem Cell Res Ther. (2019) 10:251. 10.1186/s13287-019-1369-8 31412925 PMC6693165

[36] Aragones G Suarez M Ardid-Ruiz A Vinaixa M Rodriguez MA Correig X. Dietary proanthocyanidins boost hepatic NAD(+) metabolism and SIRT1 expression and activity in a dose-dependent manner in healthy rats. Sci Rep. (2016) 6:24977. 10.1038/srep24977 27102823 PMC4840337

[37] Makide K Uwamizu A Shinjo Y Ishiguro J Okutani M Inoue A. Novel lysophosphoplipid receptors: their structure and function. J Lipid Res. (2014). 55:1986–95. 10.1194/jlr.R046920 24891334 PMC4173991

