# Supplementary material for: Targeted Lipidomics Reveal the Effect of Perchlorate on Lipid Profiles in Liver of High-Fat Diet Mice
Source: Front Nutr. 2022 Mar 14;9:837601. doi: 10.3389/fnut.2022.837601 (PMC8964020; doi:10.3389/fnut.2022.837601)
Supplement: Supplementary file 1 [file Table_1.DOCX]

Supplementary Material

**Supplementary Table 1.** Mass spectrum parameters of 34target lipid metabolites and their isotope-labeled internal standard.

| Serial No. | Compound Name | MRM transition (*m/z*) | Dephasing potential (V) | Collision energy (V) |
| --- | --- | --- | --- | --- |
| 1 | PG(16:0/16:0) | 721.4 > 255.3 | -70 | -50 |
| 2 | PG(18:2/20:3) | 795.5 > 305.2 | -70 | -70 |
| 3 | PG(16:0/18:2) | 745.5 > 279.2 | -70 | -50 |
| 4 | PG(18:0/18:1) | 775.5 > 281.3 | -70 | -50 |
| 5 | LPC(20:0/0:0) | 552.4 > 184.0 | 40 | 38 |
| 6 | LPC(20:1/0:0) | 550.4 > 184.0 | 42 | 40 |
| 7 | LPC(20:4/0:0) | 544.3 > 184.0 | 42 | 40 |
| 8 | PC(18:1/14:1) | 730.5 > 184.0 | 45 | 22 |
| 9 | PC(18:0/18:0) | 790.6 > 184.0 | 45 | 22 |
| 10 | PC(2:0/15:1) | 522.3 > 184.0 | 36 | 34 |
| 11 | PC(18:0/14:0) | 734.6 > 184.0 | 45 | 42 |
| 12 | PC(22:1/14:0) | 788.6 > 184.0 | 45 | 42 |
| 13 | PC(3:0/16:0) | 552.4 > 184.0 | 40 | 38 |
| 14 | PC(6:0/13:1) | 550.4 > 184.0 | 40 | 38 |
| 15 | PC(14:0/14:0) | 678.5 > 184.0 | 40 | 38 |
| 16 | PE(20:3/15:0) | 728.5 > 587.5 | 65 | 20 |
| 17 | PE(18:0/18:2) | 744.6 > 603.5 | 65 | 35 |
| 18 | SM(d18:1/19:0) | 745.6 > 184.0 | 65 | 30 |
| 19 | SM(d18:1/16:0) | 703.6 > 184.0 | 65 | 30 |
| 20 | SM(d15:3/24:0) | 769.6 > 184.0 | 60 | 30 |
| 21 | SM(d18:1/24:2) | 811.7 > 184.0 | 60 | 30 |
| 22 | TAG(18:0/18:3/18:1) | 900.8 > 601.5 | 60 | 38 |
| 23 | TAG(16:0/18:2/16:0) | 848.8 > 575.5 | 60 | 38 |
| 24 | TAG(18:0/18:2/18:2) | 900.8 > 603.5 | 60 | 38 |
| 25 | TAG(18:1/18:3/18:1) | 898.8 > 599.5 | 60 | 38 |
| 26 | TAG(18:0/18:1/18:4) | 898.8 > 577.5 | 60 | 38 |
| 27 | TAG(18:0/18:3/18:2) | 898.8 > 601.5 | 60 | 38 |
| 28 | TAG(16:0/18:2/18:3) | 870.8 > 575.5 | 60 | 38 |
| 29 | TAG(16:0/20:4/16:0) | 872.8 > 599.5 | 60 | 38 |
| 30 | TAG(16:0/20:3/16:1) | 872.8 > 601.5 | 60 | 38 |
| 31 | TAG(18:0/18:2/18:1) | 902.8 > 603.5 | 60 | 38 |
| 32 | TAG(16:0/20:2/16:0) | 876.8 > 603.5 | 60 | 38 |
| 33 | TAG(16:1/18:0/18:1) | 876.8 > 577.5 | 60 | 38 |
| 34 | TAG(16:1/18:3/18:1) | 870.8 > 599.5 | 60 | 38 |
| 35 | D31-PG(16:0/18:1) | 777.9 > 285.4 | -35 | -48 |
| 36 | D31-PE(16:0/18:1) | 748.7 > 281.2 | -40 | -45 |
| 37 | D31-PC(16:0/18:1) | 792.0 > 184.2 | 55 | 30 |
| 38 | C17-Sphinganine | 288.2 > 60.1 | 40 | 40 |
| 39 | D5-TAG(17:0/17:1/17:0) | 870.8>582.5 | 40 | 35 |
